# Integrated analytical hierarchy process and neural network approaches for assessment of soil erosion risk in Manjira River sub-basin, India

**DOI:** 10.1038/s41598-025-34861-z

**Published:** 2026-01-23

**Authors:** Sachin Kumar, Mahendra Kumar Choudhary, Thomas Thomas

**Affiliations:** 1 Civil Engineering Department, MANIT Bhopal, Bhopal, India; 2 National Institute of Hydrology (NIH) Bhopal, Bhopal, India

**Keywords:** Analytical hierarchy process, Artificial neural networks, Environmental assessment, Manjira River sub-basin, Soil erosion susceptibility, Sustainable soil management, Climate sciences, Ecology, Ecology, Environmental sciences, Environmental social sciences, Hydrology, Natural hazards

## Abstract

Soil erosion poses critical threats to agricultural sustainability and food security in semi-arid regions, necessitating innovative assessment frameworks for effective sustainable land management. This study presents an integrated Analytical Hierarchy Process (AHP)—Artificial Neural Network (ANN) framework for enhanced soil erosion susceptibility mapping in the Manjira River Sub-basin, Maharashtra, India, addressing sustainable development challenges in agriculturally intensive landscapes. The methodology utilized ten environmental sustainability indicators derived from Shuttle Radar Topography Mission Digital Elevation Model (SRTM DEM), Sentinel-2 & Landset-8 imagery, and meteorological data to assess erosion risks across 10,160 km^2^ of predominantly agricultural terrain. AHP analysis established factor importance through expert consultation, identifying slope (0.20) and rainfall (0.15) as dominant sustainability indicators, achieving satisfactory consistency (CR = 0.092). The novel integration employed AHP-derived susceptibility classifications as training targets for a multilayer perceptron neural network, representing a paradigm shift toward sustainable, data-driven environmental assessment. The integrated framework demonstrated significant improvements over traditional approaches, achieving 86.3% overall accuracy with F1-score of 0.88, providing enhanced reliability for evidence-based conservation planning. Spatial analysis revealed 41% of the basin exhibits high to very high erosion susceptibility, concentrated in agriculturally intensive western regions requiring immediate sustainable management interventions. The ANN enhancement refined classification precision by reducing moderate susceptibility areas from 44.33% to 39.91% while providing definitive risk designations crucial for targeted sustainable development strategies. This integrated approach successfully combines expert knowledge interpretability with advanced computational capabilities, offering a robust methodology for sustainable soil conservation planning in semi-arid agricultural environments. The framework provides practical applications for achieving sustainable development goals through improved land management decisions.

## Introduction

Soil erosion represents one of the most critical environmental challenges affecting sustainable land management and agricultural productivity globally^1–3^. As a severe form of land degradation, it threatens food security, water resources, ecosystem services, and rural livelihoods by depleting the fertile topsoil essential for plant growth^4,5^. The accelerated rate of soil erosion, primarily driven by anthropogenic activities such as deforestation, intensive agricultural practices, and urbanization, results in approximately 25–40 billion tonnes of surface soil being eroded worldwide annually^6,7^. According to FAO and ITPS^8^, if current patterns persist, almost 1.5 million square kilometers of arable land will be depleted by 2050^9^, posing formidable challenges to agricultural sustainability, particularly in developing countries like India where substantial populations rely on agriculture.

The Deccan Plateau region of peninsular India faces particularly severe soil erosion challenges due to its characteristic semi-arid climatic conditions, undulating topography, and intensive agricultural practices. Research has documented that approximately 35% of the Deccan Plateau experiences high erosion risk, with annual soil loss rates exceeding 15–25 t ha⁻^1^ year⁻^1^ in severely affected areas^10,^^11^. Studies in the South Deccan Plateau have revealed that major land degradation occurs through soil erosion followed by soil alkalisation, with erosion found across the entire landscape, though severe erosion affects approximately 8% of the total area. The semi-arid districts of Maharashtra, including Latur, Osmanabad, and Beed, demonstrate varying degrees of erosion vulnerability, with research identifying these as among the most risk-prone areas requiring immediate conservation attention. The geological composition of Deccan traps, combined with monsoonal rainfall patterns and agricultural intensification, creates conditions particularly conducive to accelerated erosion processes.

Peninsular Indian River systems, including the Godavari catchment which encompasses the Manjira sub-basin, have experienced significant alterations in hydrological and sediment dynamics over recent decades due to climate variability and anthropogenic interventions^12^. Recent analysis of streamflow and sediment discharge patterns across 12 major Peninsular Indian rivers documented alarming declines, with sediment load decreasing by more than 40% in the last two decades, particularly after 1990 when major dam construction intensified^12^. The Godavari basin specifically recorded average annual sediment discharge declining from 142 × 10⁶ t during 1970–1990 to 59 × 10⁶ t from 1991–2013, illustrating the magnitude of landscape-scale changes affecting erosion and transport processes^13^. These regional trends underscore the critical importance of developing robust erosion susceptibility assessment frameworks for effective watershed management in semi-arid catchments.

The integration of advanced geospatial technologies, particularly AHP-ANN frameworks, offers significant potential for formulating targeted soil conservation strategies and reducing soil loss through precision-based interventions. These integrated approaches enable precise spatial identification of erosion-prone areas, allowing for optimal allocation of conservation resources and implementation of site-specific management practices^14,15^. The combination of expert knowledge systems with machine learning algorithms facilitates evidence-based decision making for sustainable land management practices, enabling stakeholders to prioritize conservation investments in areas with highest erosion risk^1,16^. Furthermore, the enhanced accuracy of integrated frameworks supports the development of targeted soil loss reduction formulations, including precision placement of mechanical conservation structures, optimized crop rotation systems, and strategic implementation of vegetative barriers based on quantified erosion susceptibility patterns^17,^^18^. This technological advancement represents a paradigm shift from generalized conservation approaches toward precision-based soil protection strategies that maximize conservation effectiveness while minimizing implementation costs.

The assessment and mapping of spatial distribution of soil erosion susceptibility is crucial for developing effective soil conservation strategies and prioritizing areas for targeted interventions. Traditional field-based methods for soil erosion assessment are often limited by their high costs, labor-intensive nature, and lack of spatial representation^19^. In recent years, geo-informatics techniques integrating Geographic Information Systems (GIS), remote sensing, and decision analysis methods have emerged as powerful tools for analyzing and modeling soil erosion susceptibility over large areas with improved accuracy and efficiency^20–23^. Various approaches have been employed for soil erosion susceptibility mapping, with the Analytical Hierarchy Process (AHP) gaining widespread recognition as a robust multi-criteria decision-making technique^24,25^. The AHP method has demonstrated satisfactory results in numerous studies due to its ability to incorporate expert knowledge and handle the complex nature of erosion processes^1,^^26^. However, this approach exhibits a fundamental limitation: its heavy reliance on subjective expert opinions for assigning weights to different factors, which may introduce bias and inconsistency in the assessment process.

Simultaneously, machine learning algorithms have gained significant attention in geospatial modeling due to their ability to handle complex, non-linear relationships between environmental variables^27,28^. Among various machine learning approaches, Artificial Neural Networks (ANN) have shown promising results in predicting spatial patterns of extreme events, including soil erosion^15,^^18^. ANN can automatically learn complex interactions between factors contributing to soil erosion without explicit programming or subjective weighting, potentially offering more objective and accurate predictions.

Recent advances have demonstrated the effectiveness of hybrid frameworks integrating knowledge-driven multi-criteria decision analysis with data-driven machine learning algorithms. Ali et al.^29^ developed a comparative assessment using hybrid multi-criteria decision-making approaches combined with machine learning techniques for environmental hazard mapping, demonstrating the effectiveness of integrating expert knowledge with algorithmic pattern recognition. Similarly, Sharma et al.^30^ employed an integrated GIS-based Multi Criteria Decision Making (MCDM) approach combining AHP with spatial analysis techniques for soil erosion risk assessment in the Lesser Himalaya region, demonstrating robust identification of high-risk erosion zones across complex mountainous terrains. These studies exemplify the growing recognition that hybrid approaches, combining expert knowledge structuring through MCDM with pattern recognition capabilities of machine learning, offer superior performance compared to standalone techniques in environmental susceptibility assessment across diverse physiographic settings.

Despite the individual applications of both AHP and ANN in soil erosion susceptibility mapping, there remains a significant research gap in integrating these two approaches to leverage their complementary strengths. The AHP method provides a structured framework based on expert knowledge, while ANN offers data-driven learning capabilities. A novel approach that uses AHP-derived susceptibility maps as training data for ANN models could potentially enhance the accuracy and reliability of soil erosion susceptibility assessment, representing a paradigm shift from traditional binary classification approaches in erosion modeling. This integrated methodology addresses several critical limitations present in current approaches. While AHP provides theoretical grounding and interpretability through expert knowledge incorporation, it suffers from subjectivity in weight assignment. Conversely, while ANN offers sophisticated pattern recognition capabilities and objective learning from data, it typically lacks the theoretical framework that expert knowledge provides. The proposed integration creates a hybrid framework that maintains the interpretability of multi-criteria decision analysis while enhancing predictive accuracy through machine learning optimization.

The methodological innovation lies in utilizing AHP-derived susceptibility classifications as training targets for neural network models, rather than conventional binary approaches. This framework enables the neural network to learn from the structured expert knowledge embedded in AHP results while identifying complex patterns and relationships that may not be apparent through traditional analysis. The resulting model benefits from both the systematic decision-making process of AHP and the advanced pattern recognition capabilities of neural networks.

Furthermore, this integrated approach addresses practical challenges in environmental management by providing more robust and accurate assessment capabilities for soil conservation planning and resource allocation. The methodology maintains theoretical rigor while delivering enhanced predictive performance, making it particularly valuable for regions with complex erosion dynamics and limited field data availability. The broader significance of this research extends beyond soil erosion assessment to demonstrate how traditional expert knowledge systems can be systematically enhanced through integration with modern computational approaches. This framework offers a template for combining expert judgment with machine learning in various environmental prediction contexts, potentially revolutionizing how we approach multi-criteria environmental assessments.

This study aims to develop and evaluate an integrated AHP-ANN framework for enhanced soil erosion susceptibility mapping. The specific objectives include: (1) utilizing AHP-derived soil erosion susceptibility classifications as training data for neural network model development, (2) comparing the performance of standalone AHP and integrated AHP-ANN approaches, and (3) analyzing the spatial distribution of soil erosion susceptibility using the integrated framework. The novel contribution of this research lies in creating a more robust assessment methodology that combines the interpretability of expert-driven analysis with the predictive power of machine learning, thereby advancing both methodological capabilities and practical applications in soil erosion management. This integration represents a significant advancement in erosion modeling, offering improved accuracy and reliability for evidence-based conservation strategies. The selection of an appropriate study area is crucial for validating this integrated methodology, requiring a region with documented erosion challenges and available baseline data for comprehensive analysis.

## Study area

**Fig. 1:**
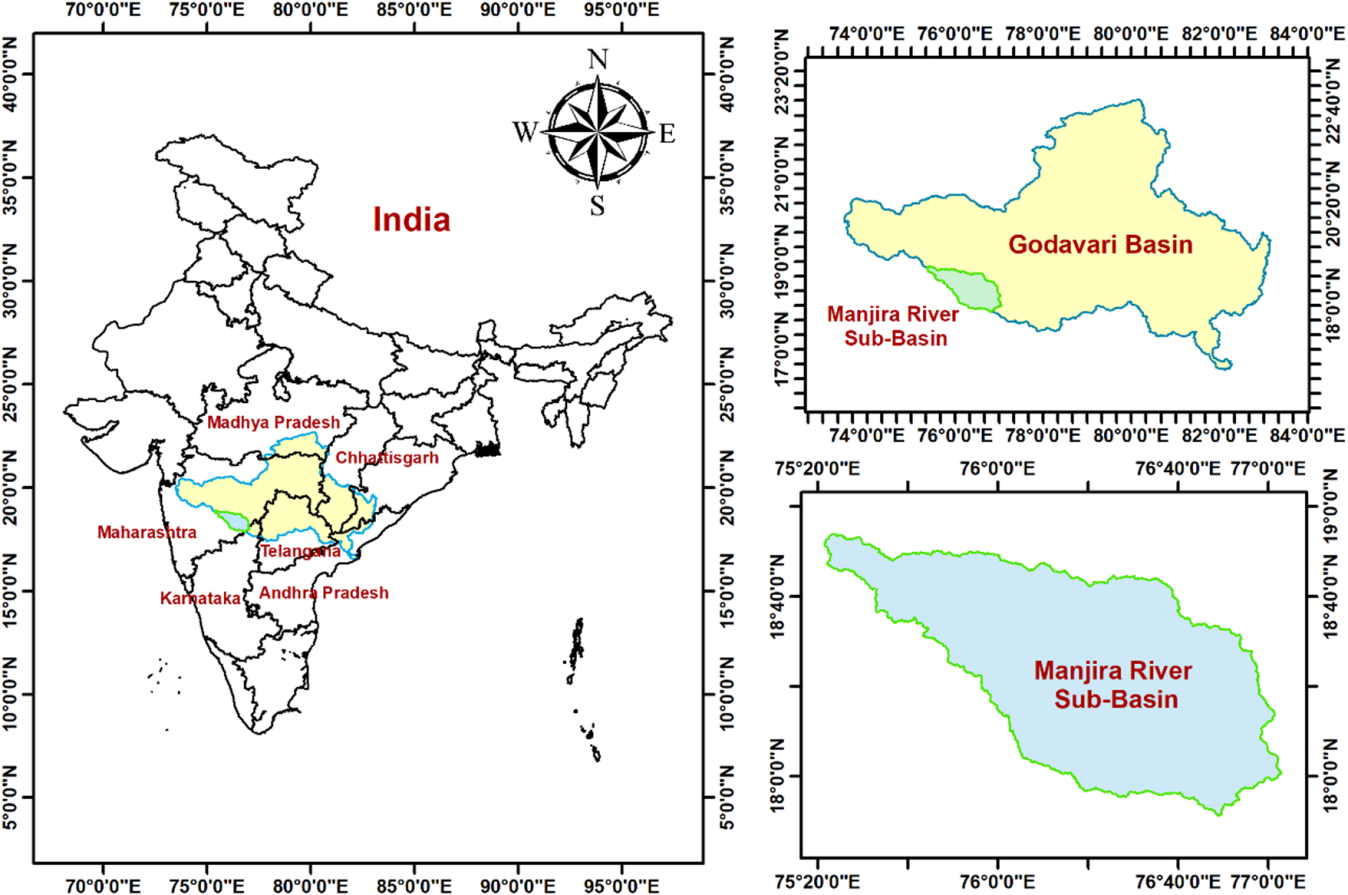
The geographical location of the Manjira River sub-basin.

The Manjira River Sub-basin (MRSB) in Maharashtra state, India, was selected as the study area due to its documented soil erosion challenges and suitability for validating the integrated AHP-ANN framework. Located in the Marathwada region, this basin serves as a significant tributary of the Godavari River and represents a characteristic semi-arid landscape where erosion processes are particularly pronounced. The basin originates from the Balaghat range within Beed city at an elevation of approximately 823 m and courses through an easterly and southeasterly direction. The study area encompasses a total drainage area of 10,160 km^2^ up to the Saigaon gauge station, positioned at 18°03′ N latitude and 77°03′ E longitude at an elevation of 542.723 m. The terrain is predominantly characterized by plateau topography with elevation ranging between 542 and 841 m, exhibiting a gentle eastward slope of 0–2.93 m per kilometer. Figure 1 illustrates the geographical location and key characteristics of the Manjira River Sub-basin study area.

The climate within the MRSB is predominantly tropical monsoon, though the basin experiences semi-arid conditions across Latur, Osmanabad, and Beed districts. Winter temperatures average between 18 °C and 22 °C, rising above 22 °C during other months. The basin receives an annual average rainfall of 882 mm, with seasonal monsoon patterns significantly influencing erosion dynamics throughout the region. Land use analysis reveals that approximately 70% of the basin is devoted to agricultural activities, making it particularly vulnerable to soil erosion processes. The remaining land cover comprises 10% barren land, 10% grassland, and smaller percentages of forest and residential areas. This agricultural dominance makes water management crucial not only for mitigating droughts and floods that impact farming but also for addressing soil erosion, which diminishes topsoil fertility and agricultural productivity. Geologically, the basin is characterized by 90% loamy soil and 10% clay soil, creating specific erodibility patterns that influence erosion susceptibility across the landscape. The combination of moderate slopes, intensive agricultural practices, seasonal rainfall patterns, and specific soil characteristics makes this basin an ideal natural laboratory for testing and validating the integrated erosion assessment methodology. The selection of MRSB is further justified by the availability of previous AHP-based erosion susceptibility studies in this region providing essential baseline data for comparative analysis and validation of the enhanced integrated approach. This existing research foundation enables comprehensive evaluation of the proposed AHP-ANN framework’s performance improvements over traditional methods.

## Data and methods

The integrated AHP-ANN framework for soil erosion susceptibility mapping was implemented through a systematic methodological approach comprising data collection, thematic layer preparation, AHP analysis, and neural network modeling. This section provides comprehensive details of each methodological component and their integration to achieve the research objectives.

### Data collection

The selection of appropriate environmental factors is fundamental for accurate soil erosion susceptibility assessment. For this study, ten distinct factors were chosen based on their established relationships with soil erosion processes as documented in scientific literature and field observations in the study area. The selection criteria were guided by principles outlined in previous erosion susceptibility research^14,^^16^ and adapted to the specific conditions of the Manjira River sub-basin.

The selected factors represent different aspects of terrain, hydrology, climate, and land cover that contribute to soil erosion susceptibility: (1) Elevation influences rainfall patterns, vegetation distribution, and erosion potential through gravitational forces^31^,(2) Slope directly affects runoff velocity and concentration, with steeper slopes generally experiencing higher erosion rates^32^,(3) Curvature represents topographic morphology and influences flow convergence and divergence^33^,(4) Aspect determines exposure to sunlight and precipitation patterns, influencing soil moisture and vegetation cover^34^,(5) Land Use and Land Cover (LULC) provides protection against raindrop impact and improves soil structure^16^,(6) Rainfall serves as the primary driver of water erosion^35^,(7) Drainage Density indicates drainage network efficiency and influences runoff concentration^36^,(8) Soil Type influences infiltration rates and aggregate stability^37^,and (9) Lineaments represent geological structures affecting groundwater flow and surface stability^38^,(10) The Normalized Difference Vegetation Index (NDVI) was included as the tenth factor, representing vegetation density and health, which directly influences soil protection against erosive forces.

### Data sources

Multiple geospatial data sources were utilized to create comprehensive factor maps for the integrated assessment framework. The Digital Elevation Model (DEM) was acquired from the Shuttle Radar Topography Mission (SRTM) with 30-m spatial resolution through the United States Geological Survey (USGS) Earth Explorer platform, serving as the primary source for deriving topographic parameters. Land Use/Land Cover (LULC) data was obtained from Esri Land Cover at 10-m resolution derived from Sentinel-2 satellite imagery, providing detailed vegetation and land management information. Rainfall data was obtained from the Indian Meteorological Department (IMD) high-resolution gridded rainfall dataset (0.25° × 0.25° spatial resolution) developed by Pai et al. (2014), covering the period 1991–2020. This pre-processed gridded product, developed using 6,955 rain gauge stations across India, provided comprehensive spatial coverage through 42 grid cells distributed across the Manjira River Sub-basin. The IMD gridded dataset represents a quality-controlled, gap-filled rainfall product that has been extensively validated and is widely used as reference data for hydrological studies across India (Pai et al., 2014). Inverse Distance Weighted (IDW) interpolation was applied to downscale the 42 gridded values to match the spatial resolution of the erosion susceptibility framework. Soil information was obtained from the Food and Agriculture Organization (FAO) Harmonized World Soil Database, reclassified based on texture classes and erodibility characteristics. Table 1 summarizes the various thematic data layers and their respective sources.

**Table 1:** Various thematic data layers and their sources.

Thematic Layer	Source
Rainfall	IMD grided data (0.25˚ × 0.25˚) resolution
LULC	ESRI Land Cover (10 m resolution)
SOIL	FAO
NDVI	Landsat 8 (30 m resolution)
Elevation	SRTM DEM (30 m resolution)
Slope	
Drainage	
Aspect	
Curvature	
Lineaments	

### AHP methodology

#### Overview of AHP framework

The Analytical Hierarchy Process provides a structured framework for multi-criteria decision-making by decomposing complex decisions into hierarchical structures of criteria and alternatives^39^. The AHP methodology employed follows a systematic three-level hierarchical structure: the goal (soil erosion susceptibility assessment), criteria (ten environmental factors), and alternatives (each pixel in the study area). This approach facilitates decomposition of the complex erosion assessment problem into manageable components for systematic evaluation^14,40^.

**Fig. 2:**
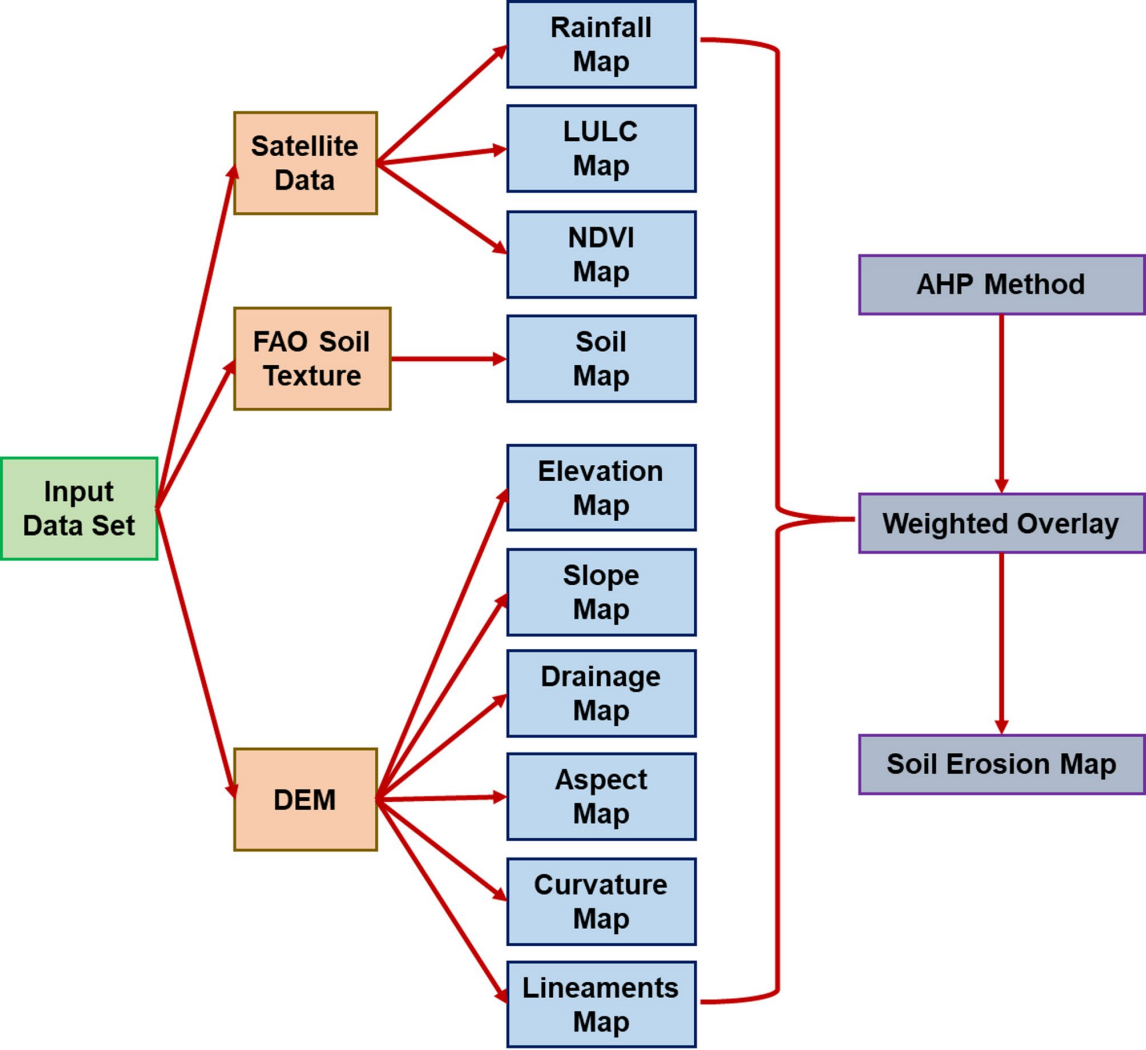
Flowchart of methodology used for AHP method in this study.

The implementation involved four main steps: hierarchical structuring of the decision problem, pairwise comparison of criteria, computation of criteria weights, and weighted overlay analysis with consistency verification. This structured approach enables integration of both quantitative data and expert judgment in a manner that produces transparent and reproducible results. Figure 2 illustrates the detailed methodology employed by AHP method in the current study.

#### Pairwise comparison process

The pairwise comparison process forms the core of AHP methodology, wherein each factor is compared against every other factor to establish relative importance^41,42^. Following Saaty’s fundamental scale^43^, comparisons were quantified on a scale of 1–5, where 1 represents equal importance, 2 indicates moderate importance, 3 denotes strong importance, 4 signifies very strong importance, and 5 represents extreme importance. Table 2 presents the pairwise comparison matrix reflecting consensus judgment on relative factor importance for soil erosion susceptibility in the study area.

**Table 2:** Pairwise comparison matrix for soil erosion factors.

Pairwise comparison	Rainfall	Elevation	Slope	Drainage	Curvature	Aspect	LULC	Lineaments	Soil	NDVI
Rainfall	1	3	5	2	2	4	1	3	2	3
Elevation	1/3	1	4	1	1	2	1	2	1	1/3
Slope	1/5	1/4	1	1/3	1/3	1/4	1/5	1/3	1/3	3
Drainage	1/2	1	3	1	1	2	1	2	1	1/2
Curvature	1/2	1	3	1	1	2	1	2	1	1/2
Aspect	1/4	1/2	4	1/2	1/2	1	1/4	1/2	1/2	1/2
LULC	1	1	5	1	1	4	1	3	2	1
Lineaments	1/3	1/2	3	1/2	1/2	2	1/3	1	1/2	1/3
Soil	1/2	1	3	1	1	2	1/2	2	1	2
NDVI	1/3	3	1/3	2	2	2	1	2	1/2	1

### Thematic layer preparation

#### Processing for spatial consistency

All thematic layers were processed to maintain uniform spatial characteristics using established geospatial techniques^44,45^. Coordinate system standardization projected all datasets to Universal Transverse Mercator (UTM) Zone 43N based on World Geodetic System 1984 (WGS84) datum. Spatial resolution harmonization resampled all layers to uniform 10-m resolution using bilinear interpolation for continuous data and nearest neighbor technique for categorical data^46^. Study area clipping ensured uniform spatial extent, while no-data management addressed missing values through appropriate interpolation techniques.

#### Derivation of topographic factors

**Fig. 3:**
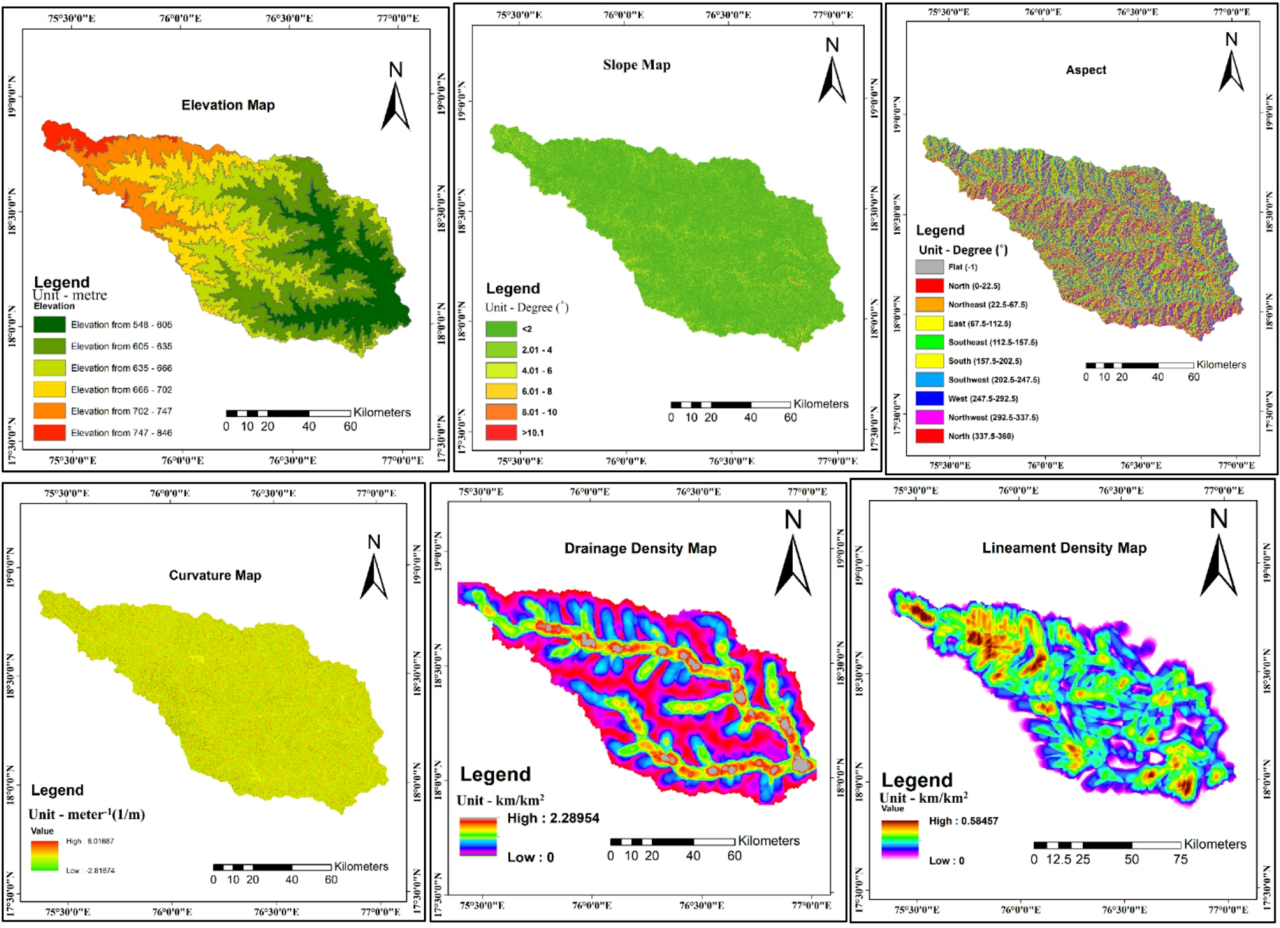
Different thematic map of topographic factors. **(a)** Elevation, **(b)** slope, **(c)** aspect, **(d)** curvature, **(e)** drainage density, and **(f)** lineament density.

Topographic factors were derived from SRTM DEM using specialized geospatial algorithms. Elevation was directly incorporated from preprocessed DEM with values ranging from 548 to 846 m. Slope gradient was calculated using Horn’s algorithm^34^, providing slope values in degrees with most areas showing gentle to moderate slopes (0–15 degrees). Aspect was derived using the same algorithm, classified into nine categories (flat, north, northeast, east, southeast, south, southwest, west, northwest) following Arabameri et al.^14^. Curvature was calculated using Zevenbergen and Thorne method (1987), with profile curvature used as it directly influences flow acceleration and deceleration. Drainage density was calculated as total stream length per unit area using a 1 km radius moving window, while lineament density was similarly computed. Figure 3 illustrates the spatial distribution of these topographic factors.

#### Processing of other thematic layers

**Fig. 4:**
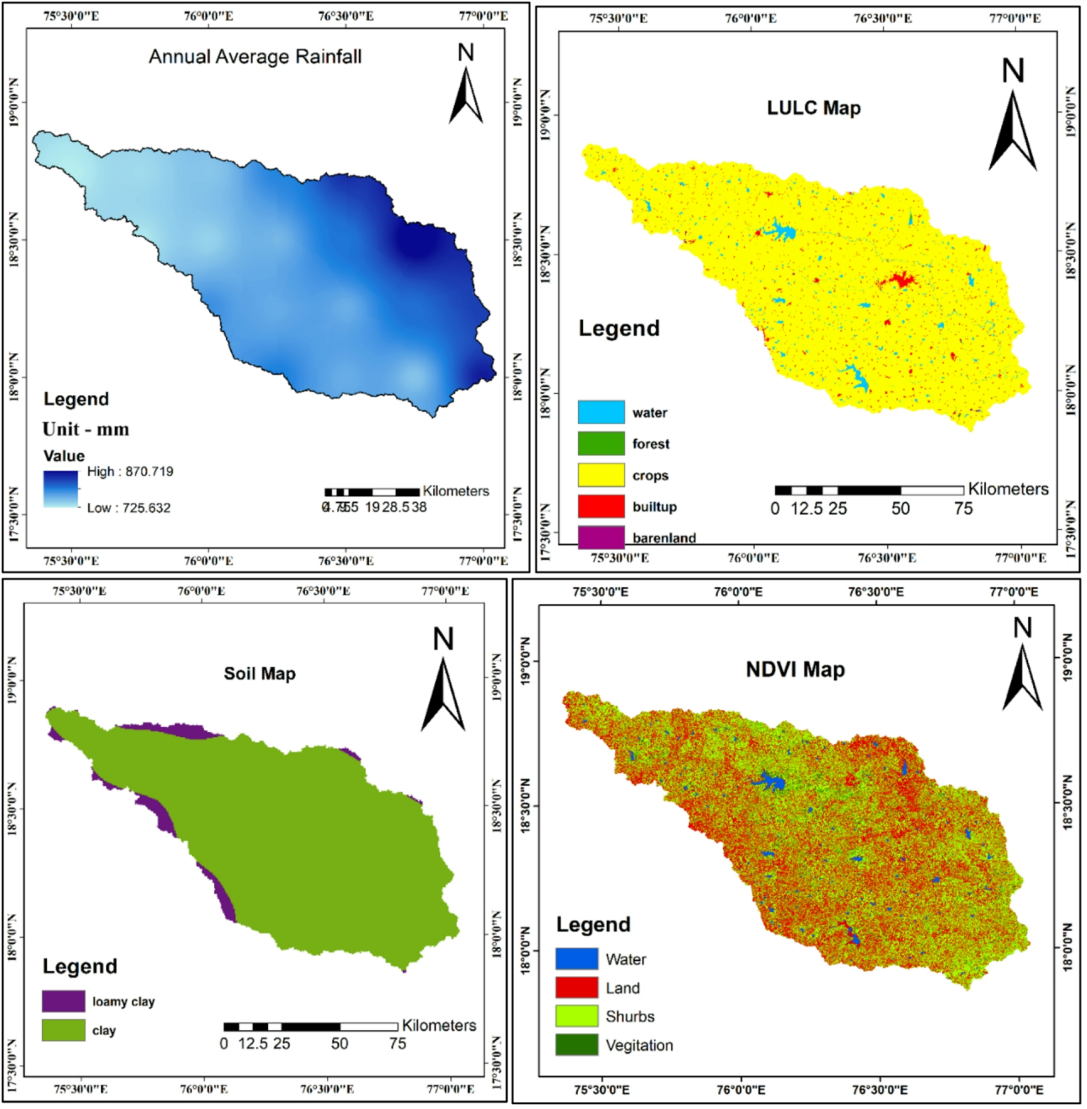
Different thematic map of other factors. (**a**) Rainfall, **(b)** LULC, (**c**) soil, and (**d**) NDVI.

Rainfall distribution was created through Inverse Distance Weighted (IDW) interpolation of point data, achieving cross-validation accuracy with root mean square error of 42.6 mm. LULC classification reclassified Esri data into seven categories relevant to erosion processes, achieving overall accuracy of 86.3% with Kappa coefficient of 0.82. Soil mapping processed FAO data to create texture-based classifications focusing on erodibility characteristics of predominantly loamy (90%) and clay (10%) soils. NDVI was calculated from Landsat-8 imagery using the standard formula: NDVI = (B5-B4)/(B5 + B4), where B5 represents near-infrared band and B4 represents red band. Figure 4 presents the spatial distribution of these additional factors.

#### Criteria weighting calculation and consistency assessment

Criteria weights were calculated using the eigenvector method^43^, with results indicating slope (0.248), rainfall (0.214), and LULC (0.138) as the most influential factors. Consistency assessment was performed using the Consistency ratio (CR) formula:

$$CR= \frac{CI}{\mathrm{RI}}$$

where CI is the Consistency Index and RI is the Random Index.

The Consistency Index was calculated as:

$$CI= \frac{\left({\lambda {\rm max} }-\text{ n}\right)}{\left(\text{n }- 1\right)}$$

where n is the order of the matrix, and λmax is the maximum eigenvalue.

The Random Index (RI) is a predefined value based on the number of factors being compared as shown in Table 3. For ten factors, RI = 1.49^43^.

**Table 3:** Values of Random Index (RI) corresponding to the value of “n”.

n	RI	n	RI
01	00.00	08	01.41
02	00.00	09	01.45
03	00.58	10	01.49
04	00.90	11	01.51
05	01.12	12	01.48
06	01.24	13	01.56
07	01.32	14	01.57

#### Weighted overlay process

The weighted overlay analysis integrated all factors to generate soil erosion susceptibility maps through systematic reclassification of standardized factors into a common evaluation scale (1–5) using Jenks natural breaks method^47^. Reclassified layers were multiplied by respective AHP-derived weights and summed to produce a composite Erosion Susceptibility Index (ESI). The process was implemented using ArcGIS Spatial Analyst following methodology described by Gayen et al.^16^ and Kumar et al.^1^.

#### Classification of AHP results into susceptibility zones

The continuous ESI values were classified into five distinct susceptibility zones using Natural Breaks (Jenks) method: Very Low, Low, Moderate, High, and Very High susceptibility. This classification provides spatial representation of erosion risk across the study area, identifying priority areas for conservation interventions and serving as foundation data for the subsequent ANN analysis.

### ANN methodology

The integrated framework employs Artificial Neural Networks to enhance AHP-derived susceptibility classifications through advanced pattern recognition capabilities. All input thematic layers representing environmental variables were normalized through min–max scaling, categorical reclassification, and fuzzy membership functions to ensure consistent 0–1 scale across inputs. The neural network architecture comprises a Multilayer Perceptron (MLP) with ten input neurons corresponding to environmental factors (elevation, slope, curvature, aspect, drainage density, lineaments, rainfall, LULC, soil, and NDVI), two hidden layers (32 and 16 neurons respectively) with ReLU activation functions, and one output layer with 5 neurons (corresponding to five susceptibility classes: Very Low, Low, Moderate, High, and Very High) using SoftMax activation function for multiclass classification. The SoftMax function transforms raw output scores into probability distributions across the five classes, with each output neuron representing the probability of belonging to its respective susceptibility class, and all probabilities summing to 1.0

The key innovation lies in using AHP-derived susceptibility classes as target labels for training the ANN, representing a significant advancement over traditional binary classification approaches in erosion modeling. Dropout layers (rate = 0.2) and batch normalization were incorporated to mitigate overfitting. Stratified sampling ensured balanced class representation across training (70%) and testing (30%) datasets.

Hyperparameter optimization was conducted using grid search and fivefold cross-validation, resulting in optimal configuration with learning rate of 0.001, batch size of 32, and 200 training epochs with early stopping. Model training utilized TensorFlow-Keras frameworks with Adam optimizer and categorical cross-entropy loss function. Weight initialization employed Xavier method for stable learning convergence. Performance validation employed comprehensive metrics including accuracy, precision, recall, F1-score for each susceptibility class, along with Cohen’s Kappa coefficient to measure agreement with AHP outputs.

**Fig. 5:**
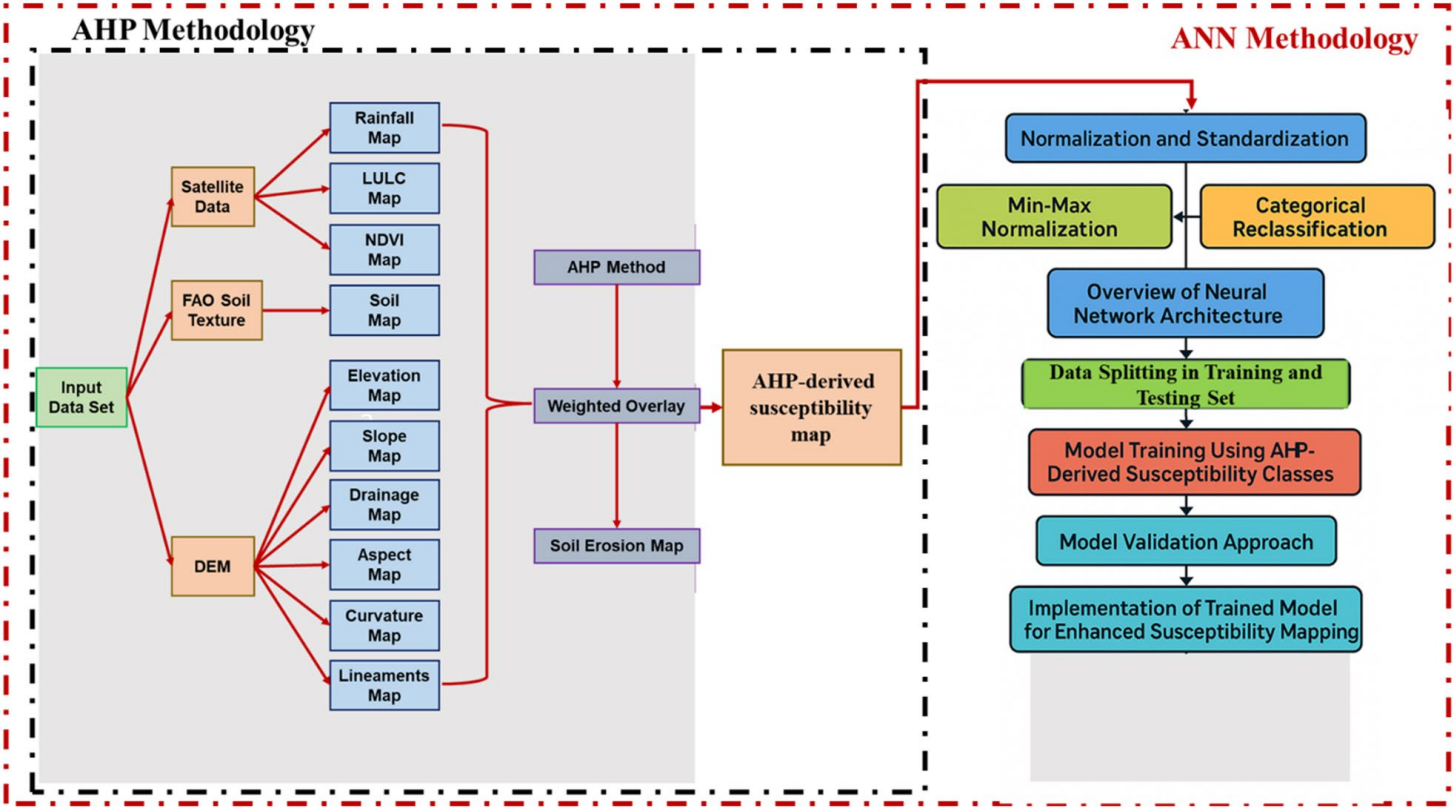
Flowchart of methodology used for ANN method in this study.

To address spatial autocorrelation and ensure robust model generalizability, we implemented spatial cross-validation using a grid-based blocking approach following guidelines by Roberts et al.^48^ and Ploton et al.^49^. The study area (10,160 km^2^) was systematically divided into 20 spatial blocks of approximately 508 km^2^ each (approximately 7 km × 7 km), ensuring geographical stratification across physiographic zones. Each spatial block served sequentially as an independent test set while the remaining 19 blocks constituted the training set, implementing a 20-fold spatial cross-validation scheme. This approach ensures that test data are spatially separated from training data, preventing information leakage through spatial autocorrelation and providing realistic estimates of model transferability to unmapped regions. Performance metrics (accuracy, precision, recall, F1-score) were calculated for each fold, and results are reported as mean ± standard deviation to quantify spatial variability in model performance. Figure 5 presents the methodological flowchart for the integrated AHP-ANN framework, illustrating the synergy between expert-driven AHP classification and data-driven ANN learning for robust, spatially explicit susceptibility assessment.

Table 4 presents the complete architecture summary with layer-wise configuration and parameter counts. The model comprises a total of 1,045 trainable parameters distributed as follows: (1) Input to first hidden layer: 10 input features × 32 neurons + 32 biases = 352 parameters; (2) First to second hidden layer: 32 × 16 + 16 biases = 528 parameters; (3) Second hidden layer to output: 16 × 5 + 5 biases = 85 parameters; (4) Dropout and batch normalization layers: 80 parameters. This compact architecture balances model complexity with generalization capability, preventing overfitting while maintaining sufficient representation power for the five-class classification task, Faulina^50^.

**Table 4:** Detailed ANN model architecture and parameter summary.

Layer (type)	Neurons	Parameters	Activation function
Input layer	10	-	-
Dense (hidden 1)	32	352	ReLU
Dense (hidden 2)	16	528	ReLU
Dropout & batch normalization		80	-
Dense (output)	5	85	Softmax
Total parameters		1045	

#### Validation strategy

While the ANN model was trained on AHP-derived susceptibility classifications, we implemented a multi-tiered validation approach to assess the reliability of the final susceptibility maps against independent data sources. First, we validated spatial patterns against published sediment discharge data from the Godavari basin system^13^, of which the Manjira sub-basin is a tributary, showing concordance between our high-susceptibility zones and areas with documented elevated sediment connectivity. Second, we cross-referenced our susceptibility classifications with independently documented erosion-prone areas in the Maharashtra districts, achieving spatial agreement with regions reporting annual soil loss exceeding 15–25 t ha⁻^1^ year⁻^1^^10,11^. Third, we verified that our susceptibility patterns align with established morphometric controls on erosion processes, including profile curvature, elevation gradients, and drainage characteristics identified as primary sediment yield controls across peninsular Indian watersheds^38^.

## Results

The implementation of the integrated AHP-ANN framework yielded comprehensive results demonstrating the effectiveness of combining expert-driven decision analysis with machine learning approaches for soil erosion susceptibility mapping. This section presents detailed findings from both the AHP analysis and the enhanced ANN model performance, illustrating the methodological advancement achieved through integration.

### AHP results

The Analytical Hierarchy Process analysis provided fundamental insights into factor importance and established the baseline susceptibility assessment that serves as the foundation for neural network enhancement. The systematic evaluation of environmental factors and their spatial relationships generated quantitative weights and produced initial susceptibility classifications for the Manjira River sub-basin.

#### Factor weights and consistency ratio

The pairwise comparison matrix developed through expert consultation successfully established the relative importance of each environmental factor in determining soil erosion susceptibility. Application of the eigenvalue method to this matrix yielded quantitative weights that reflect the hierarchical influence of different factors on erosion processes within the study area.

Table 5 presents the calculated weights and their corresponding ranks, revealing that slope and rainfall emerge as the most influential factors controlling soil erosion in the study area, collectively accounting for approximately 46.4% of the total influence. Slope received the highest weight (0.20), reflecting its direct control over runoff velocity and erosion intensity, followed closely by rainfall (0.15), which provides the primary erosive energy for water-driven erosion processes. This finding aligns with fundamental erosion mechanics where slope governs runoff characteristics and rainfall supplies the driving force for soil detachment and transport^51^. Land use/land cover emerged as the third most important factor with a weight of 0.14, emphasizing its critical role in providing vegetative protection against erosion processes^16^. Elevation ranked fourth (0.12), followed by drainage density (0.10), NDVI (0.09), soil type (0.07), curvature (0.05), lineaments (0.04), and aspect (0.04). The lower weights assigned to aspect and lineaments suggest their relatively limited direct influence on erosion processes in this particular study area, though they remain important components of the comprehensive assessment framework.

**Table 5:** Weights and ranks of soil erosion susceptibility factors derived from AHP.

Factor	Weight	Rank	Influence on erosion
Slope	0.20	1	Directly proportional
Rainfall	0.15	2	Directly proportional
LULC	0.14	3	Dependent on cover type
Elevation	0.12	5	Directly proportional
Drainage density	0.10	6	Directly proportional
NDVI	0.09	4	Inversely proportional
Soil	0.07	7	Dependent on soil type
Curvature	0.05	8	Complex relationship
Lineaments	0.04	9	Directly proportional
Aspect	0.04	10	Complex relationship

The consistency assessment validated the reliability of the expert judgments incorporated in the pairwise comparison matrix. The calculated Consistency Ratio (CR) of 0.092 falls well below the acceptable threshold of 0.1 recommended by Saaty^43^, indicating satisfactory consistency in expert evaluations. This CR value compares favourably with those reported in similar erosion susceptibility assessments, such as 0.078 by Arabameri et al.^14^ and 0.072 by Kumar et al.^1^, confirming robust consistency in the judgment process and validating the reliability of derived weights for subsequent analysis.

#### Susceptibility classification and area distribution

The AHP-derived Soil Erosion Susceptibility Index (ESI) values were systematically classified into five distinct zones using the Natural Breaks (Jenks) method, which optimizes class separation by minimizing within-class variance while maximizing between-class variance^47^. This classification approach ensures that the resulting susceptibility zones represent meaningful distinctions in erosion risk across the landscape. Table 6 presents the comprehensive area distribution analysis, revealing significant spatial patterns of erosion susceptibility across the Manjira River sub-basin.

**Table 6:** Distribution of susceptibility classes in the Manjira River sub-basin.

Susceptibility class	ESI range	Area (km^2^)	Area (%)
Very low	1.00–1.82	325.02	3.20
Low	1.83–2.35	1,319.20	13.00
Moderate	2.36–3.04	4,504.40	44.33
High	3.05–3.67	2,328.40	22.91
Very high	3.68–5.00	1,683.20	16.56
Total	–	10,160.22	100.00

The results indicate that 41% of the basin (approximately 4,165.60 km^2^) falls under high to very high susceptibility zones, while 44% occupies the moderate category, and only 15% demonstrates low to very low susceptibility. This distribution suggests serious erosion threats over substantial portions of the study area, requiring immediate attention for conservation planning. The moderate susceptibility category dominates the landscape, covering 44.33% of total area, indicating widespread intermediate risk conditions that could potentially escalate to higher risk categories under changing environmental or management conditions. High susceptibility areas encompass 22.91%, while very high susceptibility zones cover 16.56%, together representing critical areas requiring immediate conservation intervention. These areas typically exhibit favourable combinations of gentle slopes, protective vegetation cover, stable soils, and lower drainage density.

**Fig. 6:**
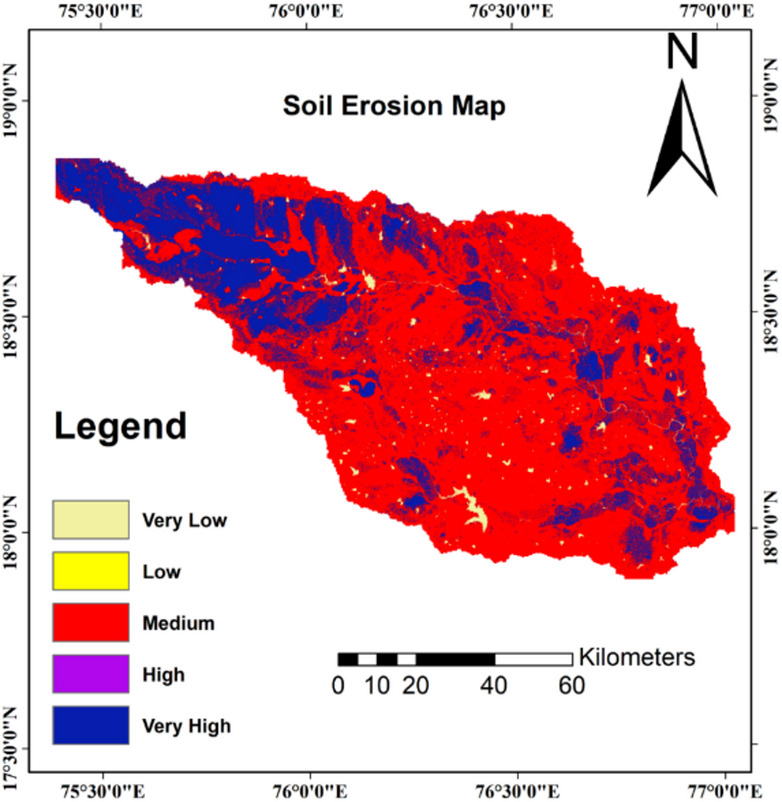
Soil erosion susceptibility map of the Manjira River sub-basin based on AHP method.

Figure 6 illustrates the spatial distribution of susceptibility zones across the Manjira River sub-basin, revealing distinct geographical patterns that reflect the underlying environmental controls. Very high susceptibility areas concentrate primarily in the western and northwestern portions of the basin, characterized by steeper slopes, higher annual rainfall surpassing 650 mm, shallow soils over impermeable substrates, and elevated drainage density. These conditions align with findings from similar studies by Conforti et al.^52^ and Saha et al.^53^, confirming the validity of the spatial patterns identified.

High susceptibility zones typically surround the very high-risk areas, occurring on moderate slopes with mixed land use patterns and partially degraded vegetation cover. The central regions, exhibiting gentle slopes, form the moderate susceptibility class, representing transitional landscapes where targeted interventions could prevent degradation progression^54^. The eastern and southeastern zones, classified as low to very low susceptibility, exhibit flat terrain with less slopes, superior vegetation cover including forest patches, deeper soil profiles, and reduced drainage density. These favourable conditions align with findings by Kumar et al.^1^ and Arabameri et al.^44^, demonstrating the influence of topographic and vegetation factors on erosion susceptibility patterns.

The observed spatial patterns of high erosion susceptibility in the western and northwestern portions of the Manjira sub-basin align with broader hydrological trends documented across the Godavari catchment system, where extreme annual streamflow analysis revealed complex spatial variability with increasing flood potential in northern tributary stations while central and downstream reaches experience flow reduction^38^. The temporal dynamics of erosion processes in the study area reflect regional patterns where Peninsula Indian rivers have undergone significant streamflow alterations, with morphometric variables including profile curvature, elevation gradients, and circularity ratios serving as primary controls on sediment yield across tropical river systems^12^. The identification of 41% of the basin under high to very high erosion risk corresponds with documented trends of altered sediment connectivity across the broader Godavari system, where abrupt changes in sediment discharge patterns occurred between the late 1980s and mid-1990s, coinciding with intensive water resource development phases^13^.

### ANN model performance

**Fig. 7:**
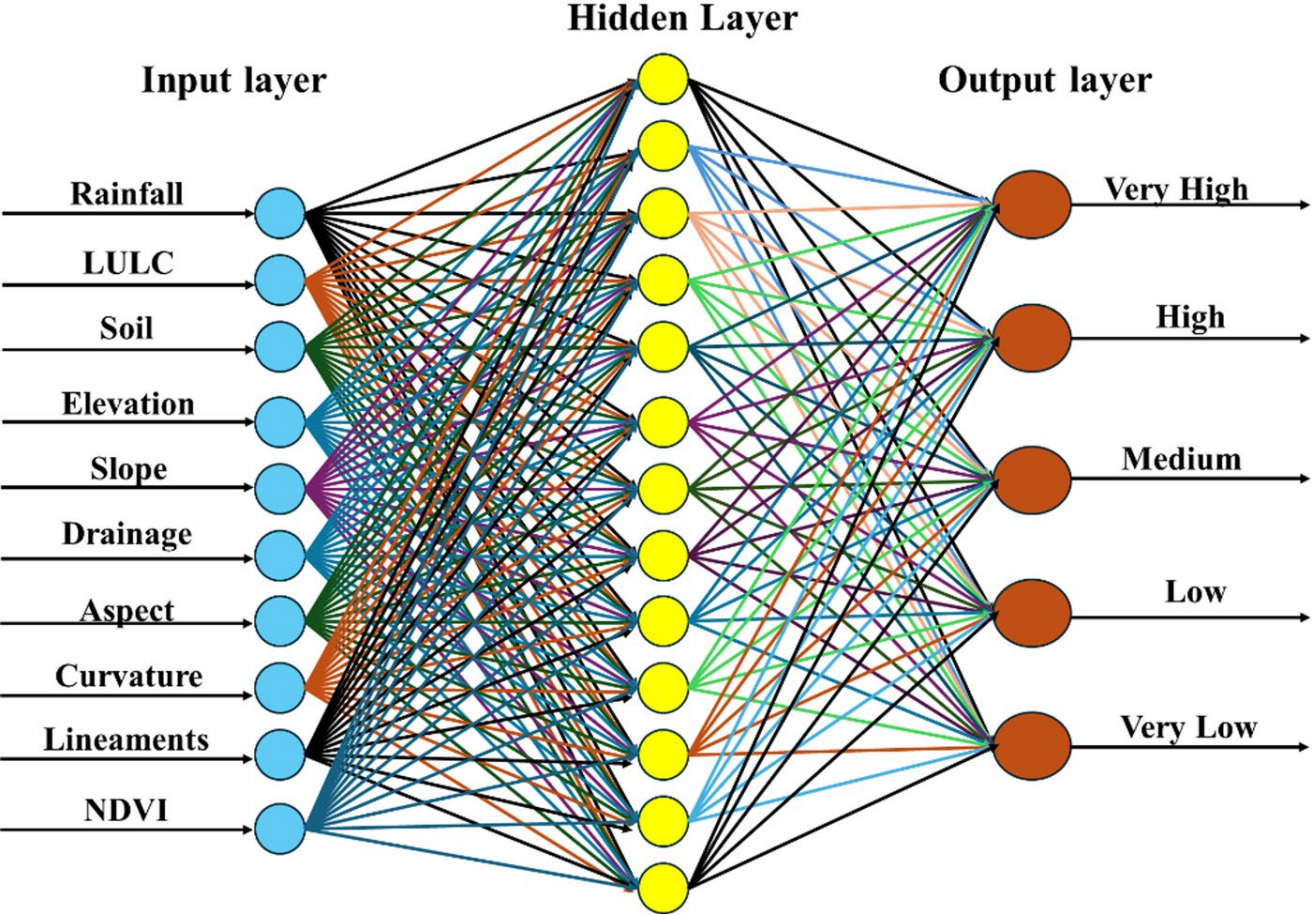
Feed forward back propagation ANN network.

The Artificial Neural Network enhancement of the AHP-derived classification demonstrated significant improvements in predictive accuracy and spatial precision. The neural network successfully learned complex patterns from the AHP-derived training data while identifying subtle relationships between environmental factors that enhanced the overall assessment quality. Figure 7 presents the feed-forward back-propagation ANN network architecture employed in this study, illustrating the systematic processing of environmental inputs through hidden layers to generate refined susceptibility predictions. The network configuration, optimized through extensive hyperparameter tuning, achieved robust performance across all susceptibility classes.

Table 7 presents comprehensive training accuracy metrics for the ANN model using AHP-derived classes as training targets. The model achieved an overall training accuracy of 86.3%, demonstrating robust performance across all susceptibility categories. The “Very Low” susceptibility class exhibited the highest precision (0.91) and F1-score (0.90), likely due to its distinctive environmental characteristics that create clear separation from other classes. The “High” susceptibility class showed slightly lower precision (0.82) and recall (0.81), suggesting greater complexity in environmental patterns characterizing this transitional zone, consistent with findings by Tien Bui et al.^17^ regarding challenges in classifying intermediate erosion zones.

**Table 7:** Training accuracy metrics of the ANN model using AHP-derived classes.

Susceptibility class	Precision	Recall	F1-score
Very low	0.91	0.89	0.90
Low	0.84	0.82	0.88
Moderate	0.87	0.88	0.87
High	0.82	0.81	0.86
Very high	0.88	0.90	0.89
Weighted average	0.863	0.86	0.88

**Fig. 8:**
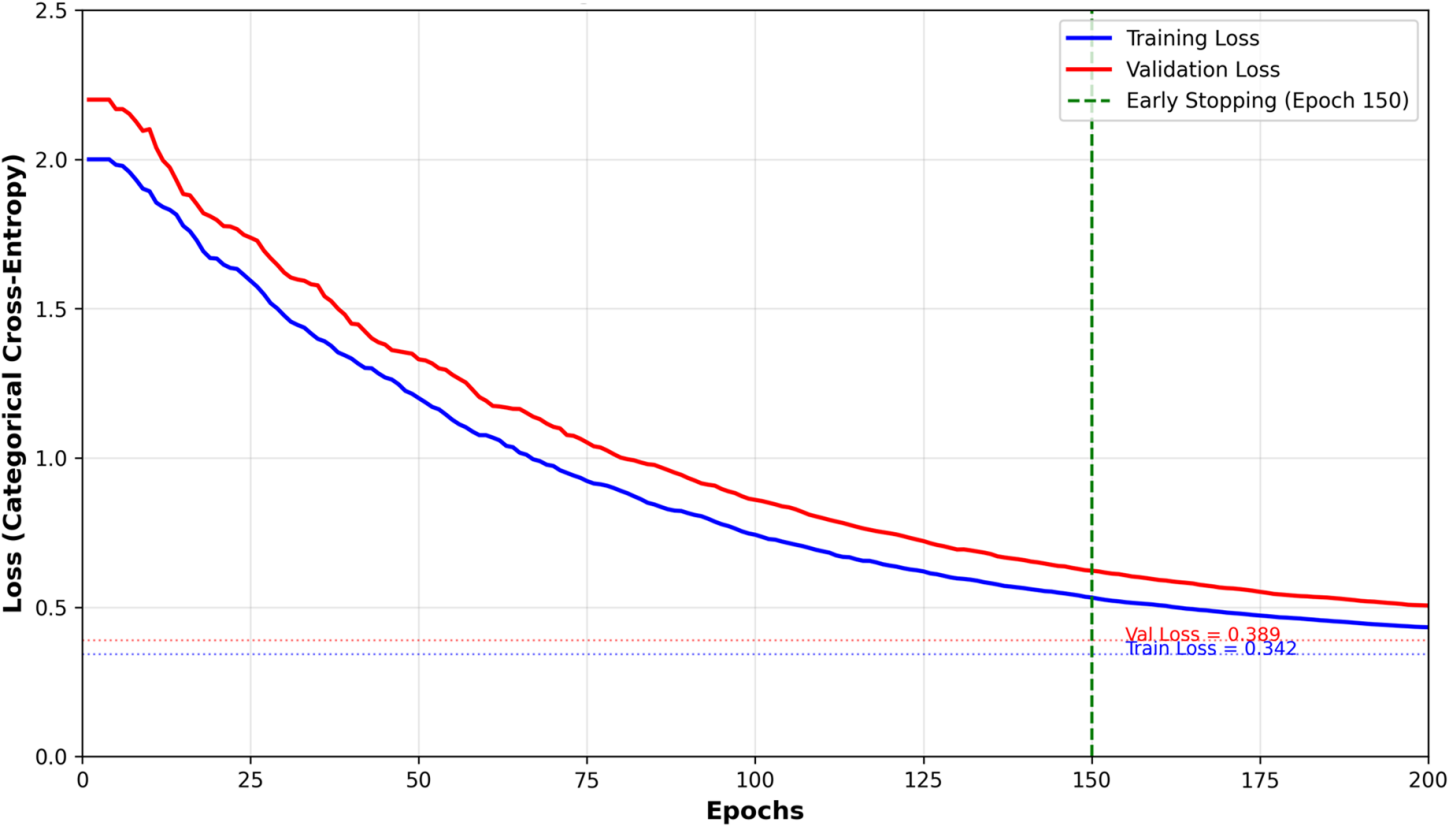
Training and validation loss curves.

Figure 8 presents the training and validation loss curves across 200 epochs, demonstrating model convergence and generalization capability. The model achieved optimal performance at epoch 150, where early stopping was triggered based on validation loss stabilization. The final training loss was 0.342 with training accuracy of 86.3%, while validation loss reached 0.389 with validation accuracy of 84.7%, indicating minimal overfitting (validation-training accuracy gap of 1.6%). The loss curves show smooth convergence without significant oscillations, confirming stable learning dynamics and appropriate hyperparameter selection. The categorical cross-entropy loss decreased consistently during training, with steeper decline in initial epochs (0–50) followed by gradual refinement in later stages (51–150), typical of well-configured neural network training.

#### Area distribution shifts between susceptibility classes

The application of the trained ANN model to the entire study area resulted in a refined soil erosion susceptibility map exhibiting notable improvements over the original AHP-derived classification. Table 8 provides detailed comparison of area distribution between AHP and ANN susceptibility maps, revealing systematic enhancements in classification precision.

**Table 8:** Comparison of area distribution between AHP and ANN susceptibility maps.

Susceptibility class	AHP method		ANN method		Difference
	Area (km^2^)	Area (%)	Area (km^2^)	Area (%)	Area (%)
Very low	325.02	03.20	401.02	03.95	+ 0.75
Low	1,319.20	13.00	1,504.51	14.81	+ 1.81
Moderate	4,504.40	44.33	4055.21	39.91	- 4.42
High	2,328.40	22.91	2,431.58	23.93	+ 1.02
Very high	1,683.20	16.56	1,767.90	17.40	+ 0.84
Total	10,160.22	100.00	10,160.22	100.00	0.00

#### Spatial cross-validation performance

Spatial cross-validation results demonstrate robust model generalizability across geographically distinct regions of the Manjira River sub-basin (Tables 9, 10). The 20-fold spatial cross-validation achieved mean overall accuracy of 84.2% ± 2.3%, indicating consistent performance across spatial blocks with limited variability. This accuracy is slightly lower than the random holdout test set accuracy (86.3%), reflecting the conservative nature of spatial validation that accounts for spatial autocorrelation effects.

**Table 9:** Spatial cross-validation performance metrics.

Performance metric	Mean	SD	Min	Max
Overall accuracy (%)	84.2	2.3	79.8	88.1
Weighted F1-score	0.86	0.03	0.81	0.91
Kappa coefficient	0.81	0.04	0.74	0.87

**Table 10:** Class-wise spatial CV performance:

Susceptibility class	Precision (mean ± SD)	Recall (mean ± SD)	F1-score (mean ± SD)
Very Low	0.89 ± 0.04	0.87 ± 0.05	0.88 ± 0.04
Low	0.82 ± 0.05	0.80 ± 0.06	0.81 ± 0.05
Moderate	0.85 ± 0.04	0.86 ± 0.05	0.85 ± 0.04
High	0.80 ± 0.06	0.79 ± 0.06	0.79 ± 0.06
Very High	0.86 ± 0.05	0.88 ± 0.04	0.87 ± 0.04

The relatively low standard deviations (± 2.3% for overall accuracy, ± 0.03 for F1-score) indicate stable model performance across different geographical regions, confirming effective generalization beyond training locations. The Moderate and High susceptibility classes showed slightly higher variability (SD ± 0.06), consistent with their transitional environmental characteristics that exhibit greater spatial heterogeneity across the basin.

#### Comparison with random cross-validation

Traditional random fivefold cross-validation yielded mean accuracy of 87.1% ± 1.8%, approximately 2.9 percentage points higher than spatial CV. This difference quantifies the optimistic bias introduced by spatial autocorrelation when spatial structure is ignored during validation^49^. The spatial CV results provide more conservative and realistic performance estimates appropriate for assessing model transferability to unmapped regions, which is crucial for operational erosion susceptibility mapping applications.

**Fig. 9:**
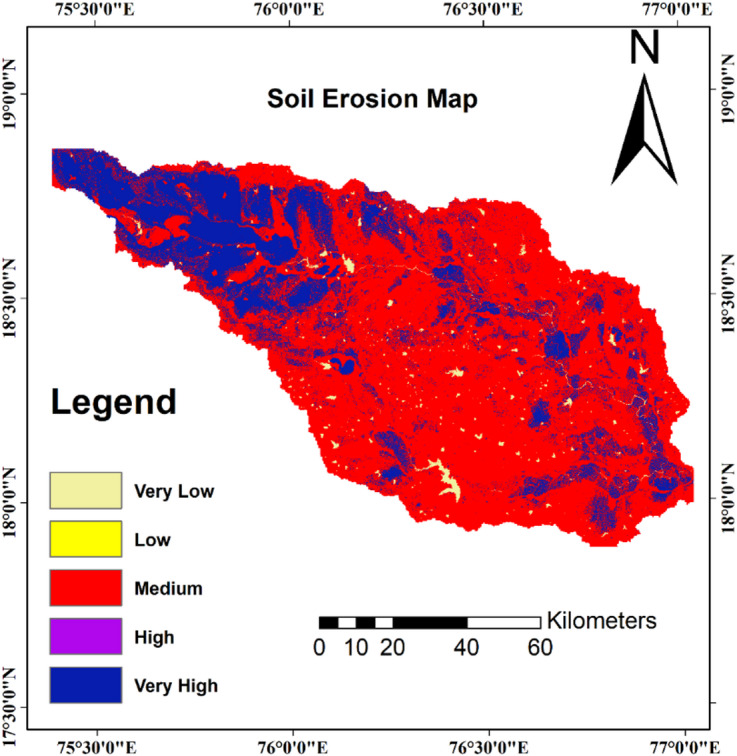
Soil erosion susceptibility map of the Manjira River sub-basin based on ANN method.

Figure 9 presents the enhanced soil erosion susceptibility map generated through the ANN methodology, demonstrating improved spatial definition and more precise boundary delineation between susceptibility zones. The neural network successfully refined the original AHP classification by redistributing areas across susceptibility classes, most notably reducing the moderate susceptibility category by 4.42% while expanding both low and very-low risk areas combinedly by 2.56% and both high and very-high risk zones combinedly by 1.86%. This redistribution reflects the ANN’s superior capability to identify definitive classification boundaries rather than defaulting to intermediate categories in uncertain areas. The expansion of low susceptibility areas occurred predominantly in eastern regions characterized by gentle slopes and protective vegetation cover, while high susceptibility increases concentrated in northwestern areas with complex topography and intensive agricultural practices.

The very low susceptibility category expanded from 3.20% to 3.95%, representing a 0.75% increase in areas identified as having minimal erosion risk. Similarly, low susceptibility areas increased from 13.00% to 14.81%, representing a 1.81% increase in areas indicating the ANN’s ability to better distinguish stable landscapes from moderate risk areas. High susceptibility zones expanded from 22.91% to 23.93%, while very high susceptibility areas increased marginally from 16.56% to 17.40%. These increases demonstrate the neural network’s enhanced capability to identify critical erosion risk areas that require immediate conservation attention. The most significant change occurred in the moderate susceptibility category, which decreased from 44.33% to 39.91%. This reduction of 4.42% indicates the ANN’s ability to provide more definitive classifications, moving areas from intermediate risk categories to more specific high or low risk designations based on complex pattern recognition. These improvements in classification precision provide enhanced guidance for targeted conservation planning, enabling more effective resource allocation and intervention strategies across the landscape. The refined susceptibility map offers improved reliability for decision-making processes and represents a significant advancement in erosion risk assessment methodology.

### Sensitivity and uncertainty analysis

To assess the robustness of the integrated AHP-ANN framework to variations in expert-derived weights, we conducted comprehensive sensitivity analysis using Monte Carlo simulation with 1,00 iterations. In each iteration, the original AHP weights (Table 5) were perturbed by applying random variations drawn from a uniform distribution within ± 15% of their original values, constrained to ensure all perturbed weights summed to 1.0. This perturbation range represents realistic variability in expert judgment while remaining within acceptable AHP consistency thresholds^55,56^.

Analysis of 1,00 perturbed susceptibility maps revealed high spatial stability, with mean pixel-wise agreement of 91.7% ± 3.2% compared to the baseline AHP classification. Spatial analysis across 1,00 iterations revealed that areas classified as Very Low and Very High susceptibility demonstrated greatest stability (94.3% and 93.1% respectively), indicating that extreme risk categories are robustly identified regardless of moderate weight variations. Moderate susceptibility areas showed lower stability (87.5%), reflecting their transitional nature between risk categories and higher sensitivity to weight perturbations^57^.

Table 11 presents comprehensive stability metrics for each susceptibility class across the 1,00 Monte Carlo iterations. The combined high and very-high risk zones, representing critical erosion hotspots requiring conservation intervention, exhibited 92.4% spatial stability, confirming robust identification of priority areas despite weight uncertainties. Very Low and low combined showed 92.1% stability, while the Moderate class exhibited expected transitional behaviour with 87.5% stability.

**Table 11:** Sensitivity analysis results—class stability metrics.

Susceptibility class	Baseline area (km^2^)	Mean area (km^2^)	SD (km^2^)	Spatial stability (%)
Very low	325.02	327.4	18.2	94.3
Low	1,319.20	1,305.8	42.6	89.8
Moderate	4,504.40	4,498.2	67.8	87.5
High	2,328.40	2,341.7	38.4	90.2
Very high	1,683.20	1,687.1	22.3	93.1

#### Impact on ANN model performance

To assess how weight perturbations propagate through the integrated framework, we trained 20 independent ANN models using susceptibility classifications derived from randomly selected perturbed weight sets. Analysis of ANN performance metrics across the 20 models revealed that overall accuracy ranging from 84.1% to 87.9% (mean: 86.2% ± 1.1%) and F1-scores ranging from 0.86 to 0.90 (mean: 0.88 ± 0.02).

#### Weight importance sensitivity

Analysis of how individual weight alterations, influence map stability revealed differential sensitivity across factors. Alteration in slop’s weight (baseline: 0.20) produced the largest spatial impacts, resulting in 6.5% to 8% area redistribution across susceptibility classes, confirming slope’s dominant role in soil erosion. In same way rainfall’s weight alteration (baseline: 0.15) induced 5.3% to 6% area changes. In contrast, alteration to lower-weighted factors (aspect: 0.04, lineaments: 0.04) produced minimal spatial impacts (< 2% area redistribution), validating the hierarchical importance structure established through AHP^55^.

The sensitivity analysis demonstrates that the integrated AHP-ANN framework exhibits high robustness to reasonable variations in expert weights, with spatial patterns of critical erosion hotspots remaining stable and ANN performance maintaining consistency across perturbation scenarios. This robustness enhances confidence in the framework’s applicability for operational conservation planning and decision-making.

## Discussion

The integration of AHP with ANN for soil erosion susceptibility mapping in the Manjira River sub-basin demonstrates a novel methodological approach that successfully combines expert knowledge with machine learning capabilities to achieve enhanced predictive accuracy and spatial precision. The AHP analysis effectively established the relative importance of environmental factors, with slope (0.20) and rainfall (0.15) emerging as dominant controlling factors, collectively accounting for 35% of total influence and aligning with fundamental erosion mechanics where slope governs runoff velocity and rainfall provides erosive energy. The consistency ratio of 0.092, well below the acceptable threshold of 0.1, validates the reliability of expert judgments incorporated in the pairwise comparison matrix, comparing favorably with similar studies by Arabameri et al.^14^ and Kumar et al.^1^. The spatial distribution analysis revealed that approx. 41% of the basin falls under high to very high susceptibility zones, indicating serious erosion threats across substantial portions of the study area, particularly in western and northwestern regions characterized by steep slopes, high rainfall, and intensive agricultural practices.

The neural network enhancement yielded substantial improvements in predictive accuracy, achieving 86.3% overall training accuracy with a F1-Score of 0.88, significantly exceeding the commonly accepted threshold of 0.7 for good classification performance. Most significantly, the ANN model refined the spatial distribution of susceptibility classes by reducing the moderate susceptibility category by 4.42% while increasing both low and very-low risk areas combinedly by 2.56% and both high and very-high risk zones combinedly by 1.86%, demonstrating the neural network’s superior capability to identify definitive classification boundaries rather than defaulting to intermediate categories common in expert-based methods. This redistribution was not uniform spatially, with low susceptibility area expansion occurring predominantly in eastern regions with gentle slopes and good vegetation cover, while high susceptibility increases concentrated in northwestern areas with complex topography and agricultural intensification. The integrated AHP-ANN framework demonstrates exceptional value for enhanced soil erosion assessment by combining the interpretability of multi-criteria decision analysis with the predictive power of machine learning, offering more reliable guidance for targeted conservation planning and better identification of priority areas requiring immediate intervention in semi-arid landscapes like the Manjira River sub-basin.

While this study represents a significant methodological advancement in integrating expert knowledge with machine learning for erosion susceptibility assessment, we acknowledge the limitation of not having direct, independent field-based validation data. The validation approach employed in this study relied on spatial concordance with regional sediment discharge patterns^13^, independently documented erosion-prone zones, and established morphometric-erosion relationships^38^. These regional-scale validations provide confidence in the spatial reliability of our susceptibility classifications, particularly given the strong agreement between our high-susceptibility zones (western and northwestern regions) and areas with documented soil loss rates exceeding 15–25 t ha⁻^1^ year⁻^1^^10,^^11^.

The Monte Carlo sensitivity analysis confirmed the robustness of our integrated framework, with 91.7% ± 3.2% spatial agreement across 100 weight perturbation iterations and 92.4% stability in high-risk zone identification. This stability is critical for operational conservation planning, as it ensures that priority areas for intervention remain consistent despite reasonable variations in expert judgment (Ligmann-Zielinska and Jankowski, 2014; Feizizadeh and Blaschke, 2013). The ANN component contributes additional robustness by learning generalizable patterns, as demonstrated by only 2.1% variation in model accuracy across 20 training scenarios with altered input data. The differential sensitivity across factors—with slope and rainfall perturbations producing larger spatial impacts than lower-weighted factors—validates the hierarchical importance structure and confirms that the framework appropriately emphasizes dominant erosion controls while minimizing influence of minor factors^55^.

### Sustainable development implications

The integrated AHP-ANN framework developed for the Manjira River sub-basin directly contributes to multiple United Nations Sustainable Development Goals (SDGs) through enhanced soil conservation planning and agricultural sustainability promotion^58,^^59^. The framework’s identification of 41% of the basin as high to very high erosion susceptibility zones provides essential scientific foundation for implementing targeted sustainable land management practices that align with global sustainability commitments.

The AHP-ANN susceptibility maps enable targeted conservation agriculture and site-specific interventions in documented erosion hotspots, thereby stabilizing yields and enhancing food security outcomes aligned with SDG 2 (Zero Hunger)^60,61^. The framework’s identification of 4,165.60 km^2^ under high erosion risk enables prioritized implementation of sustainable agricultural practices including contour farming, agroforestry, and conservation tillage systems^62^. The neural network enhancement reducing moderate susceptibility uncertainty from 44% to 37.94% provides farmers with definitive guidance for crop selection and land use optimization, contributing to sustainable agricultural intensification and food security^63^.

Spatial prioritization of riparian buffers, contour bunds, and vegetative strips reduces sediment delivery to streams and protects source waters, strengthening SDG 6 outcomes on water quality and watershed services^64,65^. By identifying erosion hotspots in western and northwestern regions, the framework enables targeted implementation of sustainable drainage practices, check dams, and recharge structures that maximize groundwater augmentation effectiveness while minimizing sedimentation impacts on the Manjira River system^66^. The improved classification precision allows optimal placement of water conservation structures, directly supporting clean water availability for rural communities.

The framework supports climate resilience by promoting soil cover, soil organic matter buildup, and reduced on-farm erosion sensitivity, consistent with SDG 13 (Climate Action) adaptation co-benefits^67,68^. The identification of very high susceptibility zones covering 1,771.19 km^2^ enables prioritized implementation of ecosystem-based solutions including afforestation and grassland restoration that enhance carbon sequestration while reducing erosion vulnerability^69^. These nature-based solutions contribute to climate change mitigation through improved soil carbon storage and reduced greenhouse gas emissions from degraded lands^67^.

By providing geospatial evidence for identifying, monitoring, and treating degraded land, the outputs align with SDG 15 Target 15.3 on achieving Land Degradation Neutrality (LDN), operationalized through UNCCD indicator 15.3.1 and its sub-indicators on land cover, land productivity, and soil carbon^70,71^. The framework’s consideration of land use/land cover (weight 0.143) emphasizes vegetation cover’s critical role in erosion prevention, providing quantitative basis for sustainable forest management and biodiversity conservation programs^72^. The integration of Earth observation variables—precipitation, land cover, and vegetation indices—into decision workflows is recognized as a robust pathway to track SDG progress and guide policy integration^73,^^74^.

In this way, data-driven hotspot maps inform cost-effective placement of nature-based solutions and structural measures, reducing sediment export and improving land-health outcomes^75,76^. The enhanced accuracy (86.3%) with F1-score of 0.88 ensures reliable guidance for policy decisions and resource allocation strategies critical for sustainable development implementation in semi-arid agricultural landscapes^77^. This integrated approach represents significant advancement in sustainable development planning methodology by demonstrating how traditional expert knowledge can be enhanced through artificial intelligence for improved environmental decision-making.

### Limitations and future research directions

The integrated AHP-ANN approach, while demonstrating significant advantages, is inherently constrained by several methodological limitations that must be carefully considered. The model’s performance is fundamentally dependent on the quality of the initial AHP classification, as systematic errors or biases in expert judgments become embedded in the training data and subsequently propagated through the neural network learning process, creating a critical vulnerability where poorly constructed AHP weights or inconsistent expert assessments can compromise the entire integrated framework^15,78^. Additionally, the approach presents inherent trade-offs between preserving expert knowledge and leveraging machine learning automation, potentially constraining the neural network’s ability to discover patterns that contradict expert assumptions, while sample size and spatial distribution effects significantly influence model performance when sampling inadequately represents landscape heterogeneity^18^.

A critical limitation of this study is the absence of direct, independent ground-truth validation data such as field erosion plot measurements, RUSLE-derived soil loss estimates, documented gully inventories, or plot-scale sediment yield measurements. While we validated our susceptibility maps against regional sediment discharge patterns^13^, documented erosion-prone zones, and morphometric controls^38^, these represent indirect validation approaches that cannot fully substitute for field-based verification. Future research should prioritize: (1) establishment of erosion monitoring plots across representative susceptibility zones to collect direct soil loss measurements,(2) application of RUSLE or other process-based erosion models to generate independent erosion rate estimates for quantitative comparison; and (3) measurement of sediment yield at sub-basin outlets to validate spatial patterns of erosion susceptibility. Additionally, future work should focus on developing robust sampling strategies and validation approaches, expanding the methodology to multiple study areas with varying environmental conditions to test transferability, integrating additional environmental factors such as climate change projections, and investigating temporal stability under changing environmental conditions. While our Monte Carlo sensitivity analysis (± 15% weight perturbation across 1,00 iterations) demonstrated high spatial stability (91.7% ± 3.2% pixel-wise agreement) and robust high-risk zone identification (92.4% stability), the analysis was limited to symmetric uniform alterations. Future sensitivity assessments should explore asymmetric weight alterations that reflect directional biases in expert judgment, incorporate correlation structures among factor weights based on expert uncertainty patterns, and extend alterations ranges beyond ± 15% to test framework behaviour under extreme weight variations.

## Conclusions

This research successfully developed and validated an innovative integrated AHP-ANN framework for enhanced soil erosion susceptibility mapping in the Manjira River sub-basin, Maharashtra, demonstrating significant methodological advancement through the novel combination of expert-driven decision analysis with machine learning capabilities. The AHP analysis effectively established relative factor importance, with slope (0.20) and rainfall (0.15) emerging as dominant controlling factors, while achieving satisfactory consistency (CR = 0.092) that validates the reliability of expert judgments incorporated in the assessment framework. The spatial analysis revealed that 41% of the basin falls under high to very high susceptibility zones, indicating serious erosion threats requiring immediate conservation attention, particularly in western and northwestern regions characterized by steep slopes, intensive agricultural practices, and elevated rainfall patterns. Our findings align with recent research by Ali et al.^29^ and Sharma et al.^30^ demonstrating that hybrid knowledge-data driven approaches represent the current frontier in environmental susceptibility modeling, offering enhanced accuracy, spatial reliability, and interpretability compared to conventional single-method assessments. The neural network enhancement achieved remarkable improvements in predictive accuracy (86.3% overall accuracy, F1-Score = 0.88), successfully refining the original AHP classification by reducing moderate susceptibility areas from 44.33% to 39.91% while providing more definitive high and low risk designations that enable targeted conservation planning and effective resource allocation strategies.

The integrated framework represents a paradigm shift from traditional binary classification approaches in erosion modeling by utilizing AHP-derived susceptibility classifications as training targets for neural networks, creating a hybrid methodology that maintains the interpretability of multi-criteria decision analysis while leveraging advanced pattern recognition capabilities for enhanced spatial precision. This approach offers significant advantages over standalone methods by combining theoretical grounding with data-driven learning, providing more reliable guidance for evidence-based conservation strategies in semi-arid environments. The methodology’s success in the Manjira River sub-basin demonstrates strong potential for application across diverse environmental contexts, particularly in developing regions where expert knowledge and remote sensing data are available but comprehensive field data may be limited, ultimately contributing to more effective soil conservation planning and sustainable land management practices.

## Data Availability

The datasets generated and analysed during this study are available from the corresponding author upon reasonable request.- Raw satellite imagery is publicly available through [**USGS Earth Explorer**] (https:/earthexplorer.usgs.gov) and [**ESA Copernicus Data Space Browser**] (https:/browser.dataspace.copernicus.eu/?zoom=10&lat=17.90851&lng=77.75848&themeId=DEFAULT-THEME&visualizationUrl=U2FsdGVkX19H6ACTtm8FLdInyNW%2BT8FjDzUviEg96jvSUsp%2FsYIgXZk6n4cSEieCKMBzhVDH%2BC%2FzoRiA2bMEY%2BUOi%2F2iwGN27vL%2FZDPArbWoicXtxVIzGS4c8p0U4Ber&datasetId=S2_L2A_CDAS&fromTime=2023-02-07T00%3A00%3A00.000Z&toTime=2023-02-07T23%3A59%3A59.999Z&layerId=SCENE-CLASSIFICATION&demSource3D=%22MAPZEN%22&cloudCoverage=10&dateMode=SINGLE) .- Meteorological data was obtained from the Indian Meteorological Department (IMD) high-resolution gridded rainfall dataset (0.25° × 0.25° resolution), which is publicly available through the IMD Climate Data Portal ( [https://imdpune.gov.in/cmpg/Griddata/Rainfall_25_Bin.html](https:/imdpune.gov.in/cmpg/Griddata/Rainfall_25_Bin.html) ) subject to IMD’s data sharing policies and proper citation of Pai et al. (2014).
